# Recent Advances in Transition-Metal-Free Late-Stage C-H and N-H Arylation of Heteroarenes Using Diaryliodonium Salts

**DOI:** 10.3390/ph14070661

**Published:** 2021-07-11

**Authors:** Thierry Besson, Corinne Fruit

**Affiliations:** Normandie University, UNIROUEN, INSA Rouen, CNRS, COBRA UMR 6014, F-76000 Rouen, France; thierry.besson@univ-rouen.fr

**Keywords:** diaryliodonium salts, C-H arylation, *N*-arylation, heteroarenes, metal-free, late-stage functionalization, eco-friendly

## Abstract

Transition-metal-free direct arylation of C-H or N-H bonds is one of the key emerging methodologies that is currently attracting tremendous attention. Diaryliodonium salts serve as a stepping stone on the way to alternative environmentally friendly and straightforward pathways for the construction of C-C and C-heteroatom bonds. In this review, we emphasize the recent synthetic advances of late-stage C(sp2)-N and C(sp2)-C(sp2) bond-forming reactions under metal-free conditions using diaryliodonium salts as arylating reagent and its applications to the synthesis of new arylated bioactive heterocyclic compounds.

## 1. Introduction

Late-stage diversification strategies that are based on heteroaromatic frameworks that contain multiple reactive sites are challenging and attractive for use in drug discovery [1,2,3]. Because small changes can have a huge impact on bioactive profile, this approach offers unique opportunities for the generation of new drug-like analogs of lead structures without extra synthetic steps or resorting to de novo synthesis [4,5,6,7,8,9,10,11,12,13,14,15,16,17]. While various reviews on C–H functionalization of heteroarenes have been already published [18,19,20,21,22], they are mainly focused on transition metal catalysis. In the quest for mild and sustainable late-stage functionalization, transition-metal-free diversification appears as a rewarding tool to build new functionalized heteroarene derivatives onto their established bioactive framework [23,24,25,26,27,28,29,30,31,32,33,34,35].

While designing new strategies that are mild, green, and metal-free and deliver valuable compounds is a challenge, metal-free diversification reactions via hypervalent iodine (III) reagents, including diaryl-λ^3^-iodane have garnered popularity [36,37,38]. In the last decade, recent progress in this area has been achieved including selective C-H and N-H arylations of heteroarenes using non-toxic and easy-to-handle diaryliodonium salts as aromatic electrophiles [39,40,41,42]. Most of the arylation reactions show high functional group tolerance and involve a symmetric diaryliodonium salt or a non-symmetric bearing an inert coupling auxiliary, also called a “dummy” group, such as TMB (2,4,6-trimethoxyphenyl), Mes (2,4,6-methylphenyl), TRIP (2,4,6-tri*iso*propylphenyl) or uracil [43,44,45,46,47,48,49,50,51,52]. This review provides an overview of the recent developments and early examples of arylating heteroarenes under metal-free conditions using acyclic diaryliodonium salts as coupling partners. The selected recent examples, since 2014, show how the metal-free catalyzed C-H and N-H bond arylation has been able to positively affect the relevant synthesis of bioactive compounds. The mechanism details, usually involving radical intermediates, are given when they are discussed by the authors but are not fully covered here. 

### 1.1. Metal-Free C-H Arylation of Heteroarenes

**Scheme 1:**
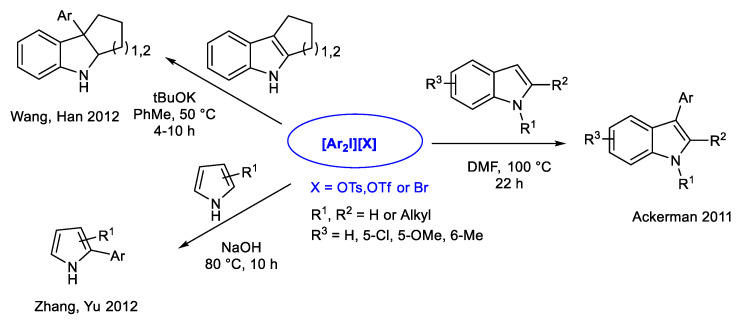
Metal-free C2-H arylation of five-membered heteroarenes using diaryliodonium salts.

In 2009, Baran first described a new method for the arylation of skatole and substituted indoles using diaryliodonium salts in the presence of *tert*-butyltetramethylguanidine (BTMG), leading to various C-3 quaternarized indole derivatives like indolines after reduction, as medicinally relevant entities [53]. The same year, Kita and co-workers developed a metal-free cross-coupling method for electron-rich heteroarenes through in situ generation of σ-heteroaryl iodine(III) intermediates [54,55]. Following these pioneering research studies, Ackermann [56], Yu and Zhang [57], and Wang and Han [58] reported direct transition-metal-free arylations of indoles, pyrroles, and carbazoles, respectively, with diaryliodonium salts (Scheme 1). The reaction was also extended to pyrazine and pyridine, but the main drawback remains the use of a large excess of the azine and no selective aryl transfer using unsymmetrical diaryliodonium salts [57]. 

**Scheme 2:**
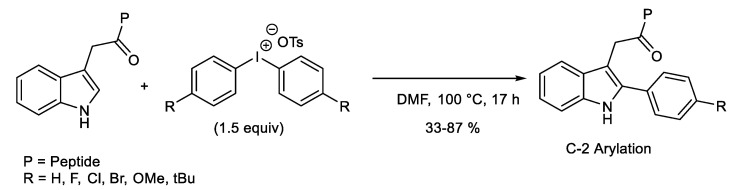
C2-H arylation of tryptophane amino acids and peptide derivatives with diaryliodonium tosylates.

In 2014, Ackerman developed the striking bioorthogonal late-stage arylation of oligopeptides derived from indole-3-acetamide [59]. The reaction took place selectively at the C2 position of the indole moiety under base-free conditions and using symmetrical diaryliodonium tosylates as arylating reagents at 100 °C in DMF (Scheme 2). This late-stage diversification is a rewarding strategy for the straightforward synthesis of analogs of tryptophan-containing peptides.

**Scheme 3:**
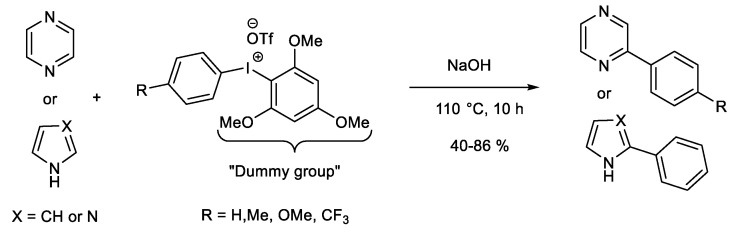
C2-H arylation of azaheteroanes using unsymmetrical aryl(TMB)iodonium triflates in the presence of NaOH.

In 2017, Kita research group explored the C2-phenylation of unprotected pyrrole as well as π-deficient azines using unsymmetrical diaryliodonium triflates in the presence of NaOH without external solvent (Scheme 3) [60]. The 2,4,6-trimethoxybenzene group (TMB) proved effective as a dummy group in favoring selective transfer of the less electron-rich and less encumbered aryl group to the heteroarene [44] and in increasing the yield of phenylated compound compared to the previous study reported by Yu and Zhang [57]. The metal-free C-H phenylation was successfully extended to imidazole, pyridine, and diazines. When the reaction is conducted with heteroarenes containing several C-H bonds as a potential point of arylation, a mixture of phenylated products was obtained. Finally, pyrazine was arylated in modest yields with *para* substituted phenyl and thiophene ligands.

**Scheme 4:**
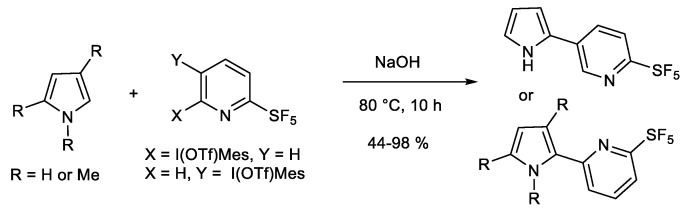
Late-stage SF_5_-pyridinylation of pyrrole derivatives.

The same year, Shibata reported a metal-free late-stage pentafluorosulfanyl (SF_5_) pyridylation of unprotected and *N*-methyl pyrroles using a novel designed electrophilic heteroaryliodonium triflates [61]. The reaction took place at the C2 position using SF_5_-pyridylmesityl-λ^3^-idoanes under the same reaction conditions reported by Kita (NaOH in pyrrole) giving the expected bi-heteroaryl products in yields ranging from 44% to 98% (Scheme 4). This attractive late-stage heteroarylation was applied to the synthesis of an SF_5_-analogue of an herbicide developed by Kumiai Chemical Ltd. and could find more applications in pharmaceutical industry.

**Scheme 5:**
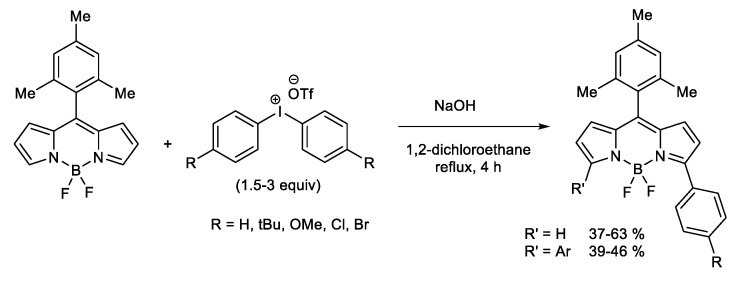
Base-mediated C-H arylation of α-free BODIPYs for the synthesis of α-arylBODIPY dyes.

**Scheme 6:**
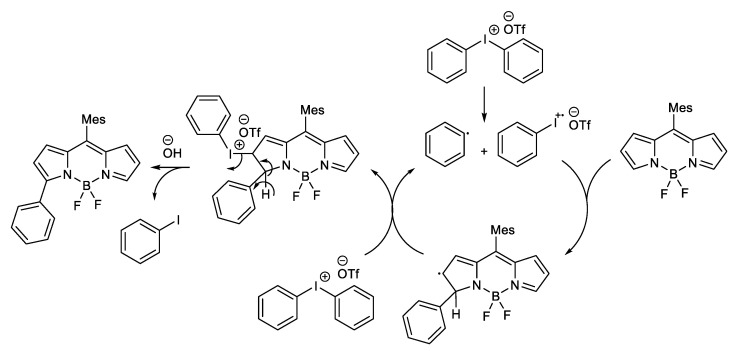
Plausible radical mechanism for the regioselective synthesis of α-arylBODIPY.

A selective base-mediated C-H α-arylation was applied by Jiao to the synthesis of mono- and diarylBODIPYs using a range of symmetrical diaryliodonium triflates (Scheme 5) [62]. Unsymmetrical diaryliodonium salts are also viable reagents and led to the selective transfer of the sterically less hindered or electron-poor aromatic moiety, in contradiction with the previously reported *ortho*-effect [63]. Owing to DFT calculations and further experiments using a radical inhibitor, the authors suggested a radical reaction pathway (Scheme 6). In addition, the calculated free energy of plausible transition states and intermediates is in agreement with the observed selectivity. The investigation of the photophysical properties showed that they are dependent on the electronic proprieties of the aryl group(s). Strong absorption and fluorescence emission were observed for the range of mono and diarylated BODIPY dyes. 

**Scheme 7:**
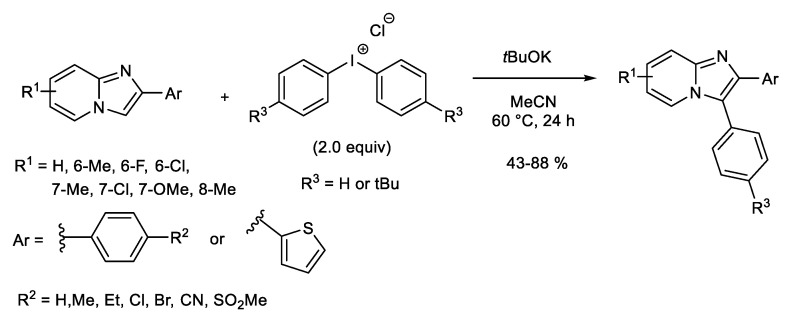
Direct late-stage C3-H arylation of various 2-arylimidazolo[1,2-*a*]pyridines using diphenyliodonium or di(*p*-*t*Bu)phenyliodonium chlorides.

In 2018, Adimurthy described the first metal-free C3-H arylation of imidazo[1,2-*a*]pyridines and imidazothiazoles using symmetrical diaryliodonium salts [64]. Eco-friendly late-stage C-H functionalization of these *N*-bearing polycyclic scaffolds has emerged as a powerful strategy for the synthesis of libraries of highly valuable compounds. Indeed, 3-substituted imidazo[1,2-*a*]pyridines are attractive compounds in drug development exhibiting various pharmacological activities as well as in optoelectronic devices [65,66,67]. The direct C3-arylations were performed in the presence of *t*BuOK (2.0 equiv) as a base in acetonitrile at 60 °C for 24 h (Scheme 7). The pivotal role of the counter anions of the diphenyliodonium was highlighted by the higher efficiency of the chloride compared to nitrate, hexafluorophosphate, or tetrafluoroborate and confirms their huge influence on reactivity [68]. 

**Scheme 8:**
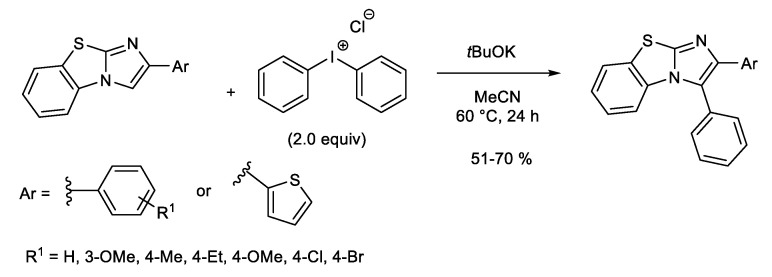
Direct late-stage C3-H arylation of various 2-arylbenzo[*d*]imidazolo[2,1-*b*]thiazoles using diphenyliodonium chlorides.

This synthetic method was successfully extended to 2-arylbenzo[*d*]imidazo[2,1-*b*]thiazoles, and the expected diarylated compounds were isolated in a 51–70% range yield (Scheme 8). The authors suggested an aryl radical intermediate based on further control experiments but without ruling out an ionic pathway involving an aryne intermediate [69].

**Scheme 9:**
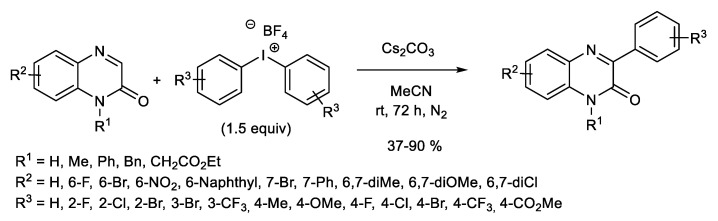
Direct late-stage C3-H arylation of quinoxalin-2(1*H*)-ones with diaryliodonium tetrafluoroborates.

**Scheme 10:**
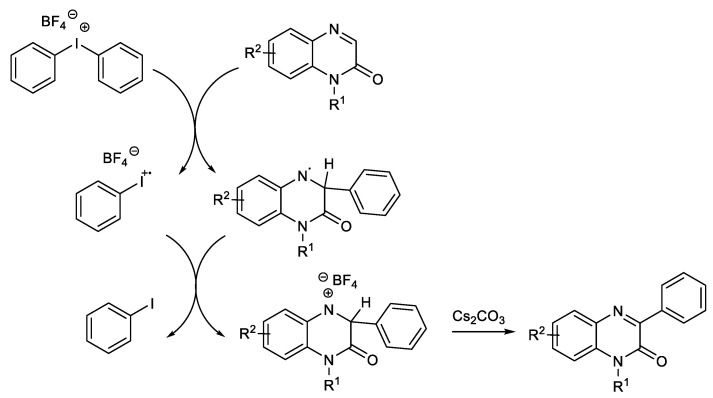
Plausible radical pathway for the synthesis of 3-aryl-quinoxalin-2(1*H*)-ones.

Because of their biological activities and chemical properties, quinoxalin-2(1*H*)-one derivatives are considered privileged scaffolds in medicinal chemistry and in materials science [70,71]. In this context, a straightforward synthesis of 3-arylquinoxalin-2(1*H*)-ones is still attractive for medicinal chemists. In 2017, Zhang developed an elegant transition-metal-free C3-H arylation of quinoxalin-2(1*H*)-one using symmetrical diaryliodonium tetrafluoroborates in the presence of cesium carbonate at room temperature (Scheme 9) [72]. Based on further experiments with a radical scavenger, the authors suggested a radical mechanism as usually reported in metal-free arylation involving high-valence iodonium salts (Scheme 10). Compared to the former reported C-H arylation of quinoxalin-2(1*H*)-ones requiring high temperature, strongly acidic conditions and/or transition metal catalysts, this strategy had several evident advantages.

**Scheme 11:**
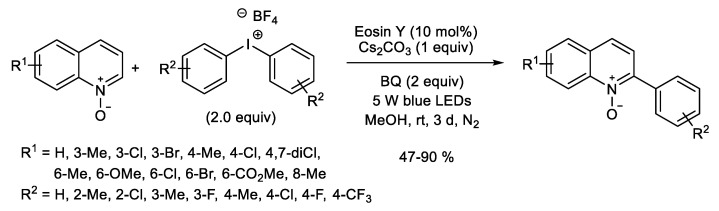
Visible-light-mediated late-stage C2-H arylation of diverse quinoline *N*-oxides using eosin Y as a photocatalyst.

**Scheme 12:**
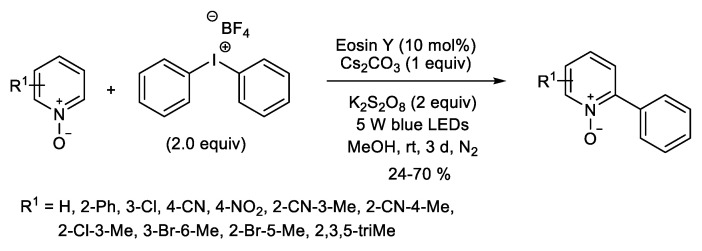
Visible-light-mediated late-stage C2-H phenylation of diverse pyridine *N*-oxides using eosin Y as a photocatalyst and K_2_S_2_O_8_ as additive.

Very recently, the first visible light-mediated arylation of pyridine and quinoline *N*-oxides using eosin Y as a photocatalyst was described by Song [73] with symmetrical diaryliodonium tetrafluoroborate as coupling reagent (Scheme 11 and Scheme 12). Support for the feasibility of this photoredox catalysis with heteroarenes is provided by recent reports [74,75,76]. Compared to the previously reported strategies of direct selective arylation of quinolone and pyridine *N*-oxides, this protocol is of benefit, requiring no transition-metal catalyst or no sensitive organometallic reagents. 

**Scheme 13:**
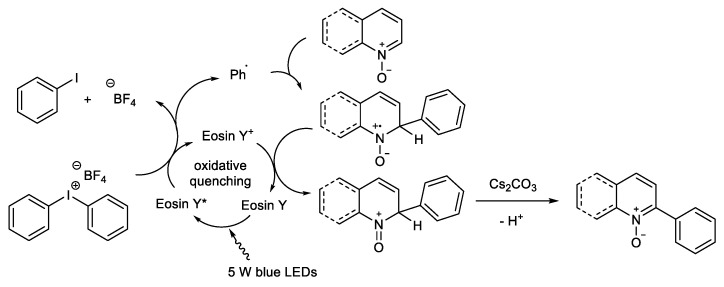
Suggested radical mechanism for the metal-free synthesis of 2-aryl quinoline and pyridine *N*-oxides.

The undertaken optimization experiments proved that the presence of 1,4-benzoquinone (BQ) as additive as well as the loadings of the diphenyliodonium salt and the photocatalyst influence the efficiency of the reaction using quinoline *N*-oxide as substrate. The reactions were performed at room temperature for 3 days under visible-light irradiation provided by a 5 W blue LED light in the presence of Cs_2_CO_3_ as a base and eosin Y to generate aryl radicals from diaryliodonium salts by a single-electron-transfer oxidizing strategy (Scheme 13). The arylation was found to be tolerant of various substituted substrates in moderate to good yields depending on the heteroarene. This late-stage C-2 arylation of these essential pharmacophores provided a practical strategy for the synthesis of attractive *N*-heterobiaryl derivatives that are widely found in pharmaceuticals and natural compounds, particularly in alkaloids [77,78,79,80,81].

### 1.2. Metal-Free N-H Arylation of Heteroarenes

**Scheme 14:**
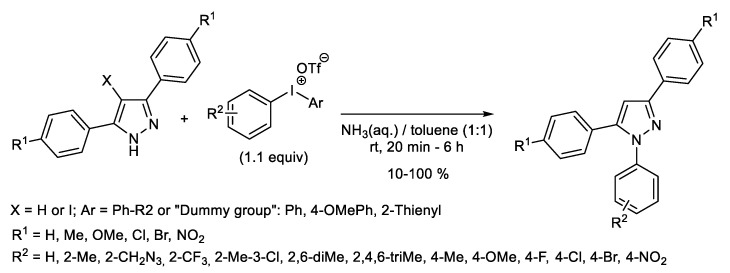
Direct late-stage *N*-arylation of substituted pyrazoles with diaryliodonium salts.

**Scheme 15:**
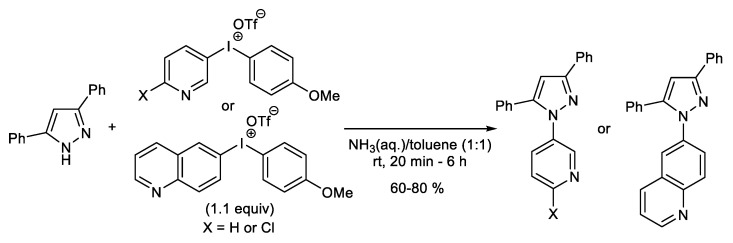
Direct *N*-heteroarylation of 3,5-diphenylpyrazole with unsymmetrical diaryliodonium triflates.

The regioselective metal-free *N*-arylation of heteroarenes using diaryliodonium salts opens new possibilities to access valuable target compounds in an eco-friendlier and cheaper process [82,83]. In addition, the *N*-arylated azoles have received considerable attention as key structural motifs of various bioactive molecules [84]. In 2012, Wang and Han reported the first transition-metal-free *N*-arylation of carbazoles promoted by diaryliodonium triflates in the presence of KO*t*Bu as base under mild conditions [58]. Few years later, notwithstanding the importance of polyaryl-substituted pyrazoles [85], Novák reported a rapid metal-free *N*-arylation of pyrazole derivatives [86]. The reactions were carried out at room temperature for 20 min or 6 h in the presence of an aqueous solution of ammonia (25 *w*/*w*%) as a base (Scheme 14). The chemoselectivity studies conducted with various unsymmetrical diaryliodonium salts bearing diverse “dummy group” highlighted “the steric effect” that favored transfer of the more hindered group but also the more electron-deficient group. Heteroaryl groups were also readily introduced on pyrazole core leading to valuable biheteroaryl scaffolds (Scheme 15). An example of an intramolecular transfer of the mesityl group was also realized starting from the corresponding pyrazolyl(Mes)iodoium triflate under the optimized conditions. 

**Scheme 16:**
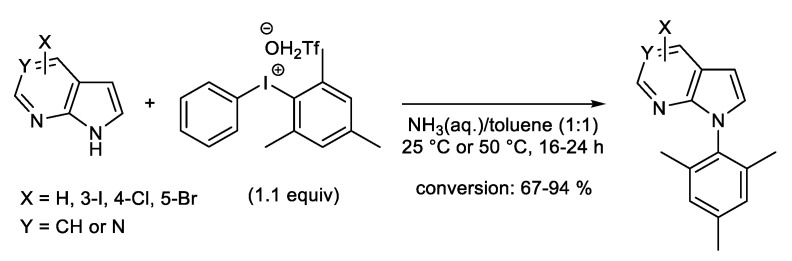
Synthesis of *N*-mesitylpyrrolo[2,3-*b*]pyridines and -pyrimidine derivatives.

**Scheme 17:**
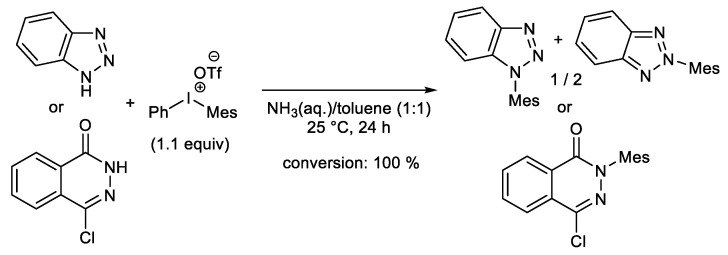
Synthesis of N-mesitylbenzo[*d*][1,2,3]triazole and 4-chloro-2-mesitylphthalazin-1(2*H*)-one.

The same group explored the mechanistic aspects of the metal-free *N*-arylation in aqueous media involving unsymmetrical diaryliodonium salts and pointed out the pivotal neighboring nitrogen effect [87]. The presence of a Lewis basic heteroatom in close vicinity to the Bronsted acid center allows the formation of a T-shape adduct through the association of the aryliodonium to the nitrogen (proximity effect). The vicinal nitrogen center brings the aryl ligand close to the N−H bond to form a favorable four-membered cyclic transition state. The optimal mechanism was predicted thanks to the comparison of the free energy profiles and is related to experimental chemoselectivity studies. Indeed, the authors conclude that the selectivity is kinetically controlled. Given a better understanding of the neighboring heteroatom effect, this computational study allowed the scope of the *N*-heteroarene to be extended to various fused-heterocycles possessing two vicinal *N*-sites without tautomerism (Scheme 16 and Scheme 17). 

**Scheme 18:**
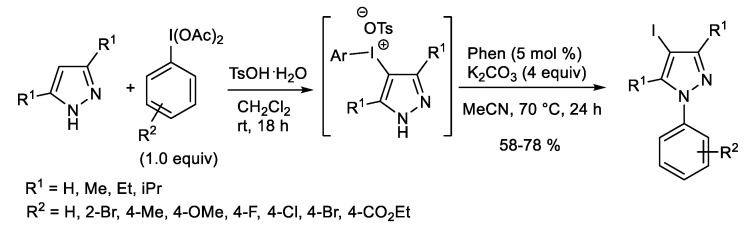
Selective iodoarylation of pyrazole derivatives via the generation of the heteroayliodonium salt as key intermediate.

In 2018, progress in the *N*-arylation of pyrazoles has also been recently achieved by Cheng and co-workers, who reported a powerful and challenging iodoarylation using the in situ generation of the corresponding heteroaryl iodonium tosylate as the stepping stone of the tandem strategy [37,88]. This metal-free, one-pot difunctionalization led to the synthesis of highly valuable 1,4-disubstitued pyrazoles. The key intermediate N-H-pyrazole-iodonium salts were generated by the reaction of pyrazole derivatives with aryliodine diacetates in the presence of TsOH·H_2_O and the sequential 1,10-phenanthroline (1,10-phen)/K_2_CO_3_-mediated arylation furnished the desired high functionalized pyrazoles (Scheme 18). 

**Scheme 19:**
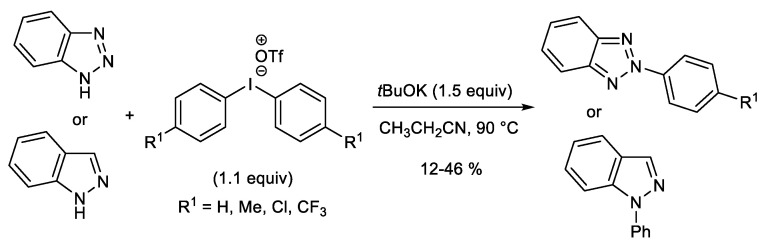
Selective *t*BuOK mediated *N*-arylation of indazole and triazole using diaryliodonium triflates as arylating reagents.

Late-stage N-H arylation of indolines was then investigated by Natchsheim to design a streamlined and rapid synthetic route to the corresponding *N*-substituted compounds as key structures in organic electronics [89,90]. The reaction was extended to benzotriazole using *t*BuOK as a base, and the selective N2-arylation was observed in moderate yield using diaryliodonium triflates in propionitrile at 90 °C (Scheme 19). One example of phenylation of indazole was also reported in low yield. 

**Scheme 20:**
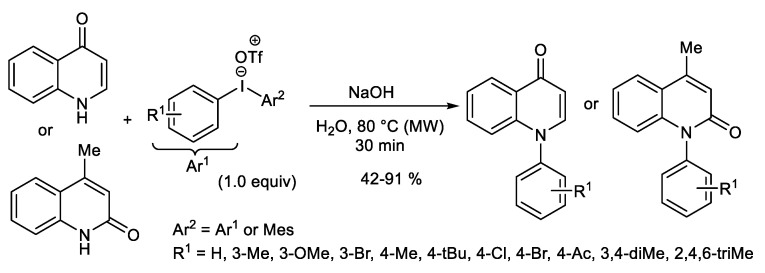
Microwave-assisted metal-free *N*-arylation of quinolin-4(1*H*)one and 4-methylquinolin-2(1*H*)-one in water.

A noteworthy strategy for the synthesis of valuable *N*-arylated quinolinones was recently accomplished by Kumar and co-workers using symmetrical diaryliodonium triflates or unsymmetrical salts bearing a mesityl as a “dummy” group [91]. *N*-arylquinolones represent a family of prominent structural motifs that are explored for their biological potential as antibacterials, antiulcers, and antitumors [92]. The appropriate quinolin-4(1*H*)-one or 4-methylquinolin-2(1*H*)-one and the diaryliodonium salt were heated in water under microwave irradiation at 80 °C for 30 min in the presence of NaOH (3.0 equiv) as a base (Scheme 20). Compared to the previously described strategy, no copper salt was needed under these mild conditions. Reaction with unsubstituted quinolin-4(1*H*)-one at C2 led selectively to the *N*-arylated compounds, whereas the *O*-arylation was obtained exclusively using the cumbersome 2-methyl and 2-phenyl derivatives, highlighting the predominant steric effect. 

**Scheme 21:**
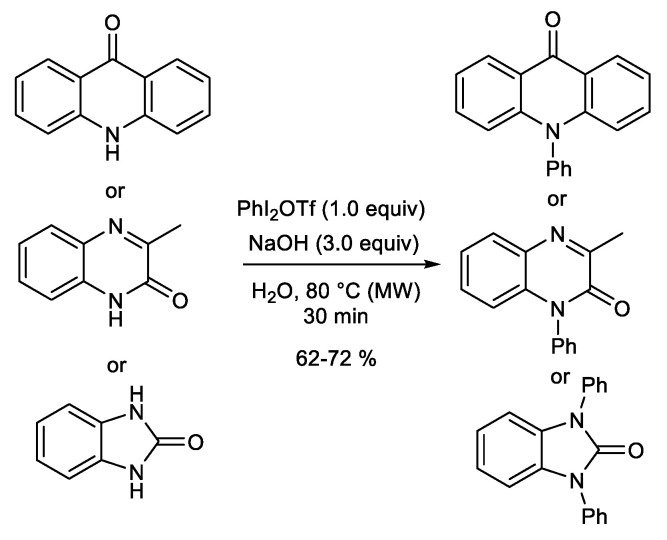
Scope of the heteroarenes for the microwave-assisted transition-metal-free *N*-arylation using diphenyliodonium triflate.

Under these optimal conditions, the scope of the heteroarene was successfully extended to acridin-9(10*H*)-one, 3-methylquinoxalin-2(1*H*)-one, and 1*H*-benzo[*d*]imidazole-2(3*H*)-one to give the corresponding *N*-phenyl compounds (Scheme 21).

**Scheme 22:**
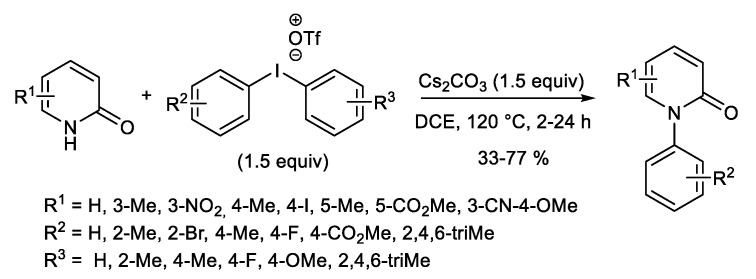
Synthesis of *N*-arylated pyridinones as major products under optimized conditions.

In 2018, driven by the design of a metal-free strategy for the *O*-arylation of 2-pyridinones, Mo and co-workers observed the competitive *N*-arylation reaction under basic conditions (tBuOK, KOH, or Cs_2_CO_3_ as the base) [93]. The reaction of substituted 2-pyridinones with diaryliodonium triflates in the presence of Cs_2_CO_3_ (1.5 equiv) in 1,2-dichloroethane (DCE) at 120 °C led to a mixture in which the *N*-arylated product was obtained in most cases as the major compound depending on the substituents on the pyridinone and the steric hindrance (Scheme 22). Various functional groups such as halides, nitro, nitrile, and ester were found to be tolerant to the reaction conditions, and some drugs or precursor of bioactive compounds with a pyridinone core were easily synthesized. Although the *N*-arylation of pyridinones was already described using copper catalysis, this approach provides an eco-friendlier complementary approach. 

**Scheme 23:**
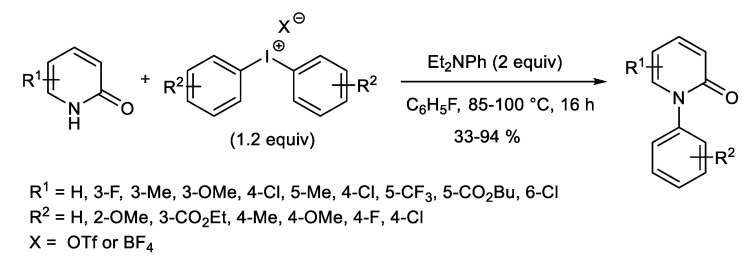
*N*-arylation of pyridin-2-ones under metal-free conditions.

Two years later, Kuriyama and Onomura disclosed a novel protocol for the selective *N*-arylation of various 2-pyridinones with diaryliodonium triflates or tetrafluoroborates (Scheme 23) [94]. This metal-free catalyzed *N*-arylation was achieved in the presence of *N*,*N*-diethylaniline as the base. The best reactivity and selectivity were observed in fluorobenzene as the solvent. This combination provides the *N*-arylated products in good yields ranging from 33% to 94% with high selectivities (>20:1). A control experiment with the 4-pyridinone furnished the expected *N*-phenylated compound with only 65% yield but with high selectivity. Both electron-donating and -withdrawing substituents on the pyridinone and diaryliodonium salts are well tolerated and led to the selective *N*-arylated compounds. Interestingly, the phenylation reaction was easily scaled up from 0.5 to 10 mmol without a decrease in yield. Further experiments on transfer selectivity were investigated using unsymmetrical diaryliodonium triflates. Whereas no electronic effect was observed, an anti-ortho effect was highlighted with diaryliodoniums with steric effect. In addition, mechanism investigations with radical scavengers suggested a mechanism not related to a single electron-transfer process. Based on Olofsson’s study on the metal-free *N*-arylation of amides, the authors proposed two pathways such as [1,2]-rearrangement as a normal ligand coupling and [2,3]-rearrangement from two possible T-shaped intermediates in equilibrium [95].

**Scheme 24:**
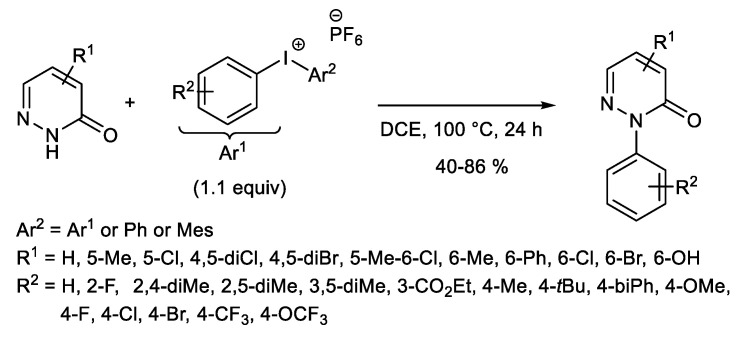
Base-free and metal-free *N*-arylation of pyridazinones with diaryliodonium hexafluorophosphates.

Progress in this area has been also recently achieved by Han and Wang [96], who described the selective metal-free *N*-arylation of 2-pyridazinone derivatives with various diaryliodonium hexafluorophosphates and its successful application to the eco-friendlier synthesis of a drug for treatment of neuropathic pain. Interestingly, the reaction proceeded in complete selectivity under base-free condition in 1,2-dichloroethane (DCE) at 100 °C for 24 h and is tolerant to various functional groups (Scheme 24). The corresponding *N*-arylpyridazinones were isolated in yields ranging from 40 to 80%, and the thienyl moiety was also readily introduced at 72% yield. Based on further experiments with radical scavengers such as TEMPO and BHT (2,6-di-*tert*-butyl-*para*-cresol), the authors suggested that an aryl radical intermediate is involved in the mechanism. 

## 2. Conclusions

Late-stage metal-free bond arylation of complex heterocycles has emerged as a sustainable and powerful tool for the synthesis of building blocks for pharmaceutical chemistry and material science. This review article summarizes the recent progress reported during the past seven years in this area using symmetrical and unsymmetrical diaryliodonium salts. Several five-membered and six-membered heteroarenes were *N*-arylated or *C*-arylated under various mild reaction conditions, including base-free or room temperature, sometimes in aqueous media. Due to their significant biological importance, the development of eco-friendlier methodologies for the synthesis of new arylated heterocycles is still of interest for the chemists’ community. With the enhancement of innovative reaction conditions, powerful late-stage diversification of valuable heteroarenes will provide an opportunity to easily access bioactive analogs to facilitate drug discovery aimed at solving existing biological problems. For example, visible-light promoted arylation reaction and photoredox catalysis open the way to the innovative and attractive synthesis of high valuable heteroarenes under mild conditions.

## Data Availability

Data sharing not applicable.
